# Usefulness of Generative Artificial Intelligence (AI) Tools in Pediatric Dentistry

**DOI:** 10.3390/diagnostics14242818

**Published:** 2024-12-14

**Authors:** Satoru Kusaka, Tatsuya Akitomo, Masakazu Hamada, Yuria Asao, Yuko Iwamoto, Meiko Tachikake, Chieko Mitsuhata, Ryota Nomura

**Affiliations:** 1 Department of Pediatric Dentistry, Hiroshima University Hospital, Hiroshima 734-8551, Japan; higechi@hiroshima-u.ac.jp (S.K.); meikosan@hiroshima-u.ac.jp (M.T.); 2 Department of Pediatric Dentistry, Graduate School of Biomedical and Health Sciences, Hiroshima University, Hiroshima 734-8553, Japan; yuriaasao@hiroshima-u.ac.jp (Y.A.); yuko-tulip@hiroshima-u.ac.jp (Y.I.); chiekom@hiroshima-u.ac.jp (C.M.); rnomura@hiroshima-u.ac.jp (R.N.); 3 Department of Oral & Maxillofacial Oncology and Surgery, Graduate School of Dentistry, The University of Osaka, Osaka 565-0871, Japan

**Keywords:** pediatric dentistry, natural language processing, artificial intelligence

## Abstract

**Background/Objectives**: Generative artificial intelligence (AI) such as ChatGPT has developed rapidly in recent years, and in the medical field, its usefulness for diagnostic assistance has been reported. However, there are few reports of AI use in dental fields. **Methods**: We created 20 questions that we had encountered in clinical pediatric dentistry, and collected the responses to these questions from three types of generative AI. The responses were evaluated on a 5-point scale by six pediatric dental specialists using the Global Quality Scale. **Results**: The average scores were >3 for the three types of generated AI tools that we tested; the overall average was 3.34. Although the responses for questions related to “consultations from guardians” or “systemic diseases” had high scores (>3.5), the score for questions related to “dental abnormalities” was 2.99, which was the lowest among the four categories. **Conclusions**: Our results show the usefulness of generative AI tools in clinical pediatric dentistry, indicating that these tools will be useful assistants in the dental field.

## 1. Introduction

Artificial intelligence (AI) is broadly defined as the ability of machines to apply human-like reasoning to problem-solving, and we have seen a rapid growth of AI in many disciplines in recent years [1]. Natural language processing (NLP), an area of research in AI, includes various methods for identifying, reading, extracting, and ultimately transforming large collections of language [2,3]. NLP techniques have gained importance in the medical field and helped in the early diagnosis of systemic diseases [4,5,6,7]. In addition, the usefulness of a state-of-the-art chatbot capable of creating natural conversations using NLP for medical consultations has been the focus of some research [8,9,10].

Dental caries and periodontitis are common oral diseases that affect a large number of people worldwide [11,12,13]. In pediatric dentistry, dental problems include malocclusion, fusion tooth, supernumerary tooth, congenital absence, and hypomineralization [14,15,16,17,18]. There are also many systemic diseases that dental professionals need to consider, such as heart disease, hemophilia, pediatric cancer, and hypophosphatasia [19,20,21,22,23]. Therefore, it is important for patients with dental problems or systemic diseases to be able to access proper information regarding dental management. Dental professionals may encounter pediatric patients whose chief complaints are dental trauma or oral habits like finger sucking or pacifier habits [24,25,26]. In addition, self-inflicted soft tissue injuries following the administration of local anesthesia can occur in pediatric patients [27]. These suggest that the field of pediatric dentistry is broad and the importance of proper knowledge for each situation.

Research using AI is also being conducted in the dental field, and the application is classified into clinical practice and dental education [28,29,30,31]. In clinical situations, AI is also used to detect caries and dental abnormalities detection, and for patient consultations [29,32,33,34]. Infectious disease pandemics can cause patients to postpone dental visits, and in some areas, a shortage of dentists can affect the difficulty of making dental visits for patients [35,36,37]. Also, early preservation is required in milk, saline solution, and even saliva in an emergency such as tooth avulsion [38]. Therefore, patient consultation using AI, especially virtual consultation, is an important topic.

Acar et al. (2024) investigated AI performance on questions regarding patients’ concerns in oral surgery and suggested the high potential of chatbots [10]. As mentioned above, there are various concerns in pediatric patients; in addition, the normal eruption of deciduous and permanent teeth into the oral cavity occurs over a broad chronologic age range [39]. Although regular dental checkups may reveal obvious dental abnormalities, patients and their parents must judge whether it is normal in current conditions [40,41]. It would benefit pediatric patients and their parents if they could obtain information about pediatric dentistry from AI and recommendations about dental visits. However, only a few reports have focused on the usefulness of AI in pediatric dental consultation for patients. In this study, we evaluated three generative AI tools to investigate whether AI can provide patients with the proper information about pediatric dentistry.

## 2. Materials and Methods

### 2.1. Ethical Consideration

Because the usefulness of generative AI was investigated in this study, ethical approval was not required and waved. No patients or medical records were involved in this study.

### 2.2. Questions Presented to the Generative AI Tools

When we searched for previous papers that focused on the reliability of responses provided via AI, the number of the questions was from 10 to 20 [10,42]. Therefore, we created 20 questions focusing on important issues in pediatric dentistry and common queries from guardians (Table 1). Questions 1–8 ask about the start or finish times of various events regarding oral management, and therefore, the three generative AI tools were able to provide the specific times of the events. Questions 9–13 focused on dental abnormalities, and Questions 14–17 focused on systemic diseases. Questions 18–20 were designed to represent questions a pediatric dentist may encounter from patients’ guardians at pediatric dental clinics.

These questions were presented in Japanese to the free versions of three generative AI in late June 2024. The same questions were always presented using the same computer on the same day to prevent the impact of updates to the AI tools. Additionally, we avoided using AI logged-in to the same account to prevent the three AI tools from affecting each other. The generative AI tools used for this study were ChatGPT 3.5 (OpenAI Global, San Francisco, CA, USA), Microsoft Copilot (Microsoft, Redmond, Washington, DC, USA), and Gemini (Google, Mountain View, CA, USA). Each question was randomly described and evaluated for this study.

### 2.3. Evaluation of Answers of the Generative AI Tools

The answers in Japanese from the generative AI tools were evaluated by six evaluators, all of whom were pediatric dentists with >5 years of clinical experience in pediatric dentistry at a university hospital, according to a previous study [21]. The evaluation criteria were the Global Quality Scale (GQS) as used in previous studies (Table 2) [10,43]. The scores were rated from 1 to 5, with 1 being the lowest and 5 being the highest. The AI response score for each question was determined by calculating the mean and standard deviation of the scores by the six evaluators.

### 2.4. Statistical Analysis

Statistical analyses were conducted using GraphPad Prism 9 (GraphPad Software Inc., La Jolla, CA, USA). A Kruskal–Wallis test for nonparametric analysis, followed by the Dunn test for multiple comparisons were used to compare between groups. Data are shown by mean ± standard deviation. Differences were considered statistically significant at *p* < 0.05.

## 3. Results

The scores of the AI responses to the 20 questions are shown in Table 3. The scores of each AI ranged from 1.00 ± 0.00 to 4.50 ± 0.55. The average of the AI scores for each question ranged from 2.61 ± 1.04 to 3.83 ± 0.71; the total average score was 3.34 ± 0.94. For the lowest AI score of 1.00, the generative AI misread the systemic disease.

**Figure 1:**
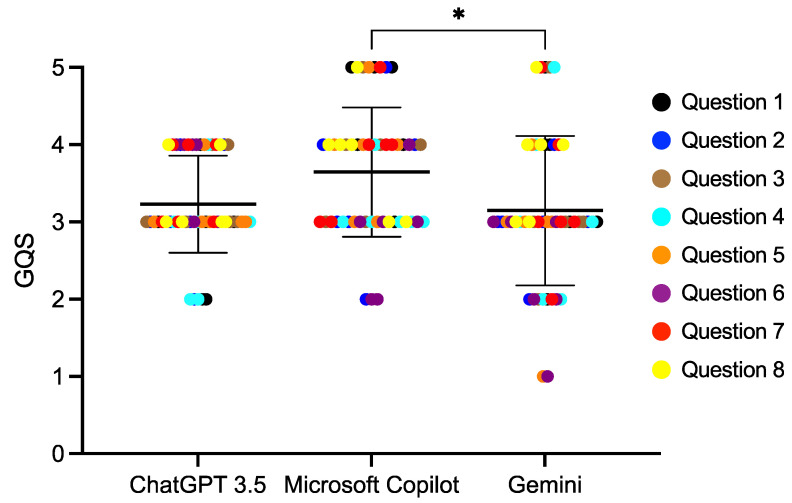
The score of the AI responses regarding age. The Kruskal–Wallis test was used for comparisons, * *p* < 0.05. Bars indicate the mean and SD.

**Figure 2:**
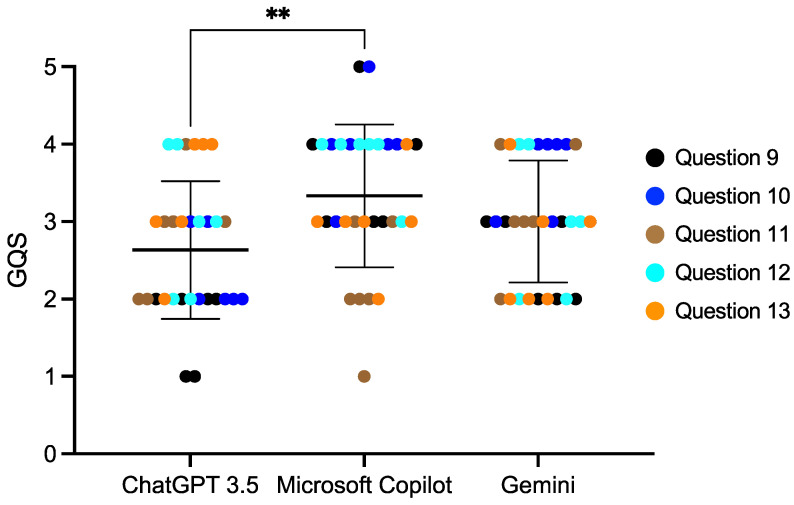
The score of the AI responses regarding dental abnormalities. The Kruskal–Wallis test was used for comparisons, ** *p* < 0.01. Bars indicate the mean and SD.

**Figure 3:**
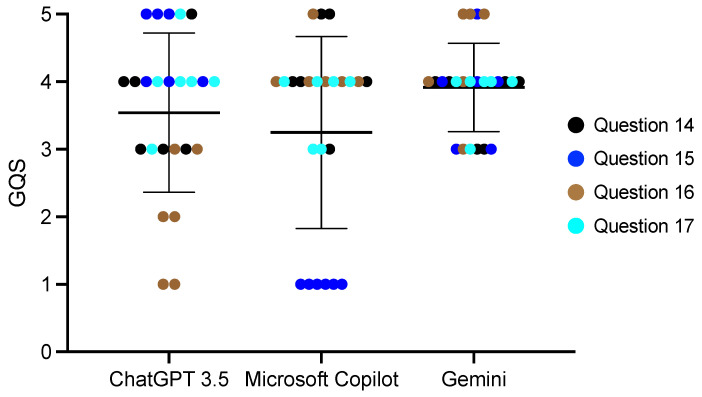
The score of the AI responses regarding systemic diseases. The Kruskal–Wallis test was used for comparisons. Bars indicate the mean and SD.

**Figure 4:**
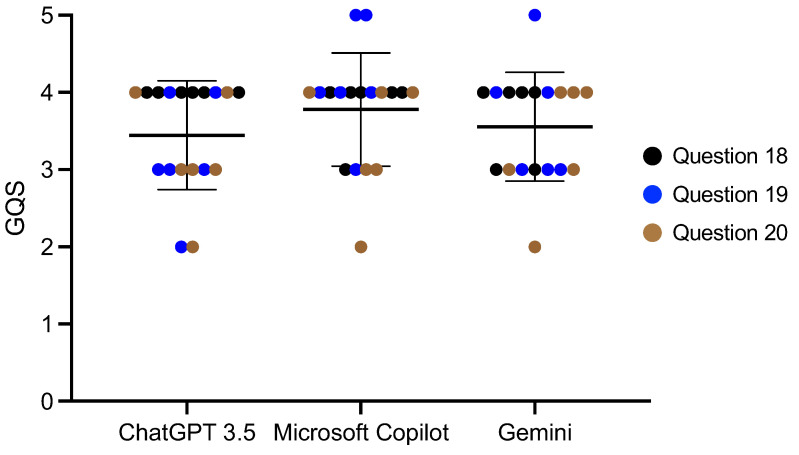
The score of the AI responses regarding consultations from guardians. The Kruskal–Wallis test was used for comparisons. Bars indicate the mean and SD.

We divided the 20 questions into four categories (Table 1) and compared the AI scores among each of the categories. For the questions regarding age, the Microsoft Copilot score was 3.65 ± 0.84, which was statistically significantly higher than Gemini (*p* < 0.01) (Figure 1, Supplementary Figure S1). Microsoft Copilot also had the highest score for the questions regarding dental abnormalities, and it was significantly different from the ChatGPT 3.5 score (*p* < 0.01) (Figure 2, Supplementary Figure S2). However, there were no differences in the responses to the questions regarding consultations from guardians (Figure 3 and Figure 4, Supplementary Figures S3 and S4).

For the responses regarding consultations from guardians, the total average score was 3.59 ± 0.71, which was the highest among the four categories, followed by the questions regarding systemic diseases and age. For the responses to the questions regarding dental abnormalities, the total average score was 2.99 ± 0.91, which was the lowest among the four categories.

## 4. Discussion

In this study, we evaluated how accurately ChatGPT, Microsoft Copilot, and Gemini answered medical questions. Screenshots of representative responses from each AI tool are shown in Supplementary Figure S5. Microsoft Copilot’s score was significantly higher than that of Gemini in responses regarding age, and it was significantly higher than that of ChatGPT 3.5 in responses regarding dental abnormalities. In response to the question “At what age should I start brushing my child’s teeth?”, Microsoft Copilot recommended starting as soon as teeth erupt, and using toothpaste once the child is able to rinse their mouth. Gemini also answered that after the primary teeth have erupted; however, it recommended starting the use of toothpaste before tooth eruption and after eruption, to use a toothpaste that does not contain fluoride. The Japanese Society of Pediatric Dentistry recommends the use of fluoride toothpaste, with careful use regarding the amount, after the primary teeth eruption, which may have influenced the evaluation [44]. In addition, in the response to the question regarding neonatal teeth, Microsoft Copilot correctly explained the definition, whereas ChatGPT 3.5 answered about neonatal teeth and congenital dental abnormalities and did not mention the definition, resulting in a difference in the evaluation scores. On the other hand, in response to the question regarding systemic diseases, Microsoft Copilot’s score was 1.00, which was the lowest because it answered about diabetes when asked about hemophilia. In this study, the responses to each item were evaluated, and when the answers included incorrect information in a lot of information, the evaluation was low. In the future, it will be necessary to compare cases where limitations such as the number of characters are set.

We found that although the AI responses had a certain degree of accuracy, several important issues were identified. First, the AI responses were based on general medical knowledge and showed relatively high accuracy for basic medical information [45]. For example, the AI tools provided reliable information for questions related to general knowledge, such as symptoms of common diseases, preventive measures, and standard treatments. When accompanied by specific numbers, the results were generally consistent with the expert knowledge of pediatric dental specialists. This is likely due to the fact that AI learns based on large data sets [46]. Ozgor et al. (2024) also reported that analyzing data from numerous resources provided a higher accuracy rate for ChatGPT’s answers [47]. However, not all the information on the Internet is accurate. Cetin et al. (2023) investigated English videos about Coronary artery bypass grafting on YouTube™, and reported that YouTube™ English videos have low quality and reliability [48]. In addition, Borges do Nascimento et al. (2022) reported that the prevalence of health-related misinformation on social media ranged from 0.2% to 28.8% [49]. It is important for AI to obtain information from the correct resources.

AI’s limitations became apparent for questions related to complex medical issues that require specialized judgment and specific advice tailored to individual patient situations. For example, it became clear that individualized treatment strategies for specific medical conditions and answers that reflect the latest research findings need to be validated by experts. AI learns based on the existing data, which limits its ability to respond to the latest information and individual cases. Massey et al. (2023) investigated the performance of ChatGPT on orthopedic assessment examinations and reported that both ChatGPT-3.5 and GPT-4 performed better on text-only questions than questions with images [50]. It is reported that the diagnostic performance of GPT-4 and GPT-4V-based ChatGPTs did not reach the performance level of either radiology residents or board-certified radiologists in challenging neuroradiology cases [51]. These results indicate that it may be currently difficult for generative AI to correctly diagnose based on patient information and examination findings. On the other hand, GPT-4 passed the Japanese Medical Licensing Examination, whereas GPT-3.5 failed the examination, suggesting GPT-4′s rapid evolution in Japanese language processing [52]. As the accuracy of AI improves in the future, this weakness may be improved.

AI answers may be ambiguous, and users may have trouble interpreting them. In particular, there is a risk of users misunderstanding if medical terminology or complex concepts are not fully explained. However, because answers from AI usually do not include the sources, we found that there were situations where it was difficult to determine how certain the information was unless one had expertise in the field. This point needs to be improved to increase the transparency and explanatory nature of AI responses.

We also found that there was a risk of problems not only in the output of information from AI tools, but also in the input to properly read information from the questions. In the present study, one AI misread a systemic disease and gave a wrong answer, resulting in the lowest score of 1.00. In clinical situations, the patient may suffer harm when misinterpretation by AI leads to an answer giving incorrect information. The answers by AI are written in text; therefore, it is important for users to verify that the AI reads and answers the questions correctly. To make appropriate use of generative AI tools, in addition to efforts to make AI output appropriate information, it is necessary to explore ways to ensure AI tools read the information properly [53].

We also considered the ethical aspects of AI. When AI provides medical information, it is important to ensure that the information is not misleading [54]. In particular, answers that promote self-diagnosis or self-treatment should be avoided, and users should always be encouraged to seek the opinion of a medical professional. When AI is used in the medical field, its responsibilities and limitations must be clearly defined [54].

AI is considered a promising technology to support non-invasive tasks in pediatric dental clinical practice, such as helping parents with their children’s oral hygiene habits and assisting general dentists in diagnosis [55,56,57,58,59]. You et al. reported an AI model for detecting plaque in primary teeth and achieved clinically acceptable performance compared to experienced pediatric dentists [56]. Kılıc et al. concluded that an AI model that detects the number of pediatric primary teeth in panoramic radiographs is a promising tool for the automatic charting of panoramic radiographs [57]. General dentists are not necessarily familiar with pediatric dental knowledge and diagnosis. Although our results require further refinement for the clinical use of AI, the application of AI may support general dentists in their understanding and diagnosis of pediatric dentistry.

AI research in clinical pediatric dentistry has many promising applications that might change pediatric practice in the coming years [60]. On the other hand, the ongoing collaboration between dental professionals, researchers, and technologists will be essential in harnessing the full potential of AI to transform pediatric dental care [61]. By accumulating large datasets and integrating and analyzing them, AI may learn clinically valuable information related to pediatric dentistry and improve the accuracy of its answers.

Overall, while generative AI tools can be useful in providing medical information, it is important to recognize their limitations and risks and to use them appropriately [62]. Future research and development must take a comprehensive approach that includes ethical considerations and methods to improve the accuracy and reliability of AI.

This study has some limitations. First, in accordance with previous reports, we used the free versions of generative AI in the present study [63,64,65]. Therefore, the results may be different if we use the paid version. In addition, the results may vary depending on the timing of the survey or the type of AI because AI has improved every day. We will investigate the change in AI by conducting similar surveys again in the future. Second, referring to the previous report [43], the 20 questions were based on the author’s personal experience and lacked standardization. We also did not investigate the scoring variability among the six evaluators. Although our study selected the evaluators based on the criteria that they had a certain amount of clinical experience, we did not calculate the Kappa value or provide evaluator training. Additional surveys are needed to simplify the evaluation criteria or investigate the inter-rater reliability in the future. Lastly, 20 questions were presented in Japanese, and the answers in Japanese were evaluated. The competence of generative AI may vary depending on the language. The advantage of evaluating in one’s native language is that it allows for a detailed understanding by evaluators. On the other hand, conducting research in English has the advantage of comparing countries or regions. We will conduct additional research to assess the difference in language.

## 5. Conclusions

To our knowledge, this is the first study to examine the usefulness of generative AI in pediatric dentistry. We found that the three generative AI tools tested provided reliable responses to many questions regarding pediatric dentistry, suggesting the usefulness of AI in clinical dental situations. However, extremely low AI scores were found for a few questions, and there were categories with significant score differences between the AI tools. Thus, generative AI has weaknesses, and it is important to understand the characteristics when using generative AI tools. Further research is needed to develop the clinical application of generative AI in dentistry.

## Figures and Tables

**Table 1:** Questions and categories used in this study (translated from Japanese).

**Age** At what age do teeth start erupting? At what age is weaning completed? At what age should I start brushing my child’s teeth? At what age should a child go to the dentist? At what age should a child use toothpaste? At what age do primary teeth start to fall out? Until what age should I continue brushing my child’s teeth? At what age should orthodontic treatment be started? **Dental abnormalities** 9. A neonatal tooth is present. Is there anything I should be careful about? 10. A fused tooth is present. Is there anything I should be careful about? 11. Supernumerary tooth is present. Is there anything I should be careful about? 12. A hypomineralized tooth is present. Is there anything I should be careful about? 13. Congenital absence of permanent tooth is noted. Is there anything I should be careful about? **Systemic diseases** 14. My child has a heart disease. Is there anything I should be careful about during dental treatment? 15. My child has hemophilia. Is there anything I should be careful about during dental treatment? 16. The primary tooth of my 4-year-old child fell out. Is it a sign of illness? 17. My child has pediatric cancer and is receiving chemotherapy. Is there anything I should be careful about in the oral cavity? **Consultations from guardians** 18. A permanent tooth fell out when my child fell down. Is there anything I should be careful about? 19. My 2-year-old child sucks their finger. Is there anything I should be careful about? 20. My child received dental caries treatment with local anesthesia. Is there anything I should be careful about?

**Table 2:** Global Quality Scale score description used in this study.

Score 1	Poor quality, poor flow of the site, most information missing, and not at all useful for patients
Score 2	Generally poor quality and poor flow, some information listed but many important topics missing, and of very limited use to patients
Score 3	Moderate quality, suboptimal flow, some important information is adequately discussed but others poorly discussed, and somewhat useful for patients
Score 4	Good quality and generally good flow, most of the relevant information is listed, but some topics are not covered, and useful for patients
Score 5	Excellent quality and excellent flow, and very useful for patients

**Table 3:** Evaluation of AI responses to 20 questions.

Question	ChatGPT 3.5	Microsoft Copilot	Gemini	Average
1	2.83 ± 0.75	4.33 ± 0.82 *	3.50 ± 1.05	3.56 ± 1.04
2	3.17 ± 0.75	3.50 ± 1.05	3.00 ± 0.63	3.22 ± 0.80
3	3.33 ± 0.52	3.83 ± 0.75	3.17 ± 0.98	3.44 ± 0.78
4	2.83 ± 0.75	3.17 ± 0.41	3.17 ± 1.17	3.06 ± 0.80
5	3.17 ± 0.41	3.83 ± 0.75	2.83 ± 0.98	3.28 ± 0.83
6	3.67 ± 0.52	2.83 ± 0.75	2.33 ± 0.82 *	2.94 ± 0.87
7	3.50 ± 0.55	3.83 ± 0.75	3.33 ± 1.03	3.56 ± 0.78
8	3.33 ± 0.52	3.83 ± 0.75	3.83 ± 0.75	3.67 ± 0.69
9	1.67 ± 0.52	3.67 ± 0.82 **	2.50 ± 0.55	2.61 ± 1.04
10	2.33 ± 0.52	4.00 ± 0.63 **	3.67 ± 0.52 *	3.33 ± 0.91
11	2.83 ± 0.75	2.17 ± 0.75	3.17 ± 0.75	2.72 ± 0.83
12	3.00 ± 0.89	3.83 ± 0.41	3.00 ± 0.89	3.28 ± 0.83
13	3.33 ± 0.82	3.00 ± 0.63	2.67 ± 0.82	3.00 ± 0.77
14	3.67 ± 0.82	4.17 ± 0.75	3.67 ± 0.52	3.83 ± 0.71
15	4.50 ± 0.55	1.00 ± 0.00 **	3.83 ± 0.75 ^†^	3.11 ± 1.64
16	2.00 ± 0.89	4.17 ± 0.41 *	4.33 ± 0.82 **	3.50 ± 1.29
17	4.00 ± 0.63	3.67 ± 0.52	3.83 ± 0.41	3.83 ± 0.51
18	4.00 ± 0.00	3.83 ± 0.41	3.67 ± 0.52	3.83 ± 0.38
19	3.17 ± 0.75	4.17 ± 0.75	3.67 ± 0.82	3.67 ± 0.84
20	3.17 ± 0.75	3.33 ± 0.82	3.33 ± 0.82	3.28 ± 0.75
Total	3.18 ± 0.90	3.51 ± 1.00	3.33 ± 0.89	3.34 ± 0.94

Data are shown as the mean ± SD. The total mean ± SD were calculated using the average of the 20 questions. Statistical comparisons were performed on each question number using the Kruskal–Wallis test. * *p* < 0.05 and ** *p* < 0.01 versus ChatGPT 3.5; ^†^ *p* < 0.05 versus Microsoft Copilot.

## Data Availability

The data are available from the corresponding author upon reasonable request.

## Supplementary Materials

The following supporting information can be downloaded at: https://www.mdpi.com/article/10.3390/diagnostics14242818/s1, Figure S1: The score of AI responses regarding age. (A) Question 1, (B) Question 2, (C) Question 3, (D) Question 4, (E) Question 5, (F) Question 6, (G) Question 7, (H) Question 8. Kruskal-Wallis test was used for comparisons, * *p* < 0.05. Bars indicate mean and SD. Figure S2: The score of AI responses regarding dental abnormalities. (A) Question 9, (B) Question 10, (C) Question 11, (D) Question 12, (E) Question 13. Kruskal-Wallis was used for comparisons, * *p* < 0.05, ** *p* < 0.01. Bars indicate mean and SD. Figure S3: The score of AI responses regarding systemic diseases. (A) Question 14, (B) Question 15, (C) Question 16, (D) Question 17. Kruskal-Wallis was used for comparisons, * *p* < 0.05, ** *p* < 0.01. Bars indicate mean and SD. Figure S4: The score of AI responses regarding consultations from guardians. (A) Question 18, (B) Question 19, (C) Question 20. Kruskal-Wallis was used for comparisons. Bars indicate mean and SD. Figure S5: Screenshots of representative responses from each AI tool (accessed on 26 November 2024). (A) ChatGPT 3.5, (B) Microsoft Copilot, (C) Gemini.
