# Supplementary material for: Usefulness of Generative Artificial Intelligence (AI) Tools in Pediatric Dentistry
Source: Diagnostics (Basel). 2024 Dec 14;14(24):2818. doi: 10.3390/diagnostics14242818 (PMC11674453; doi:10.3390/diagnostics14242818)
Supplement: Supplementary file 1 [file diagnostics-14-02818-s001.zip › diagnostics-3263625-supplementary.pptx]

## Slide 1
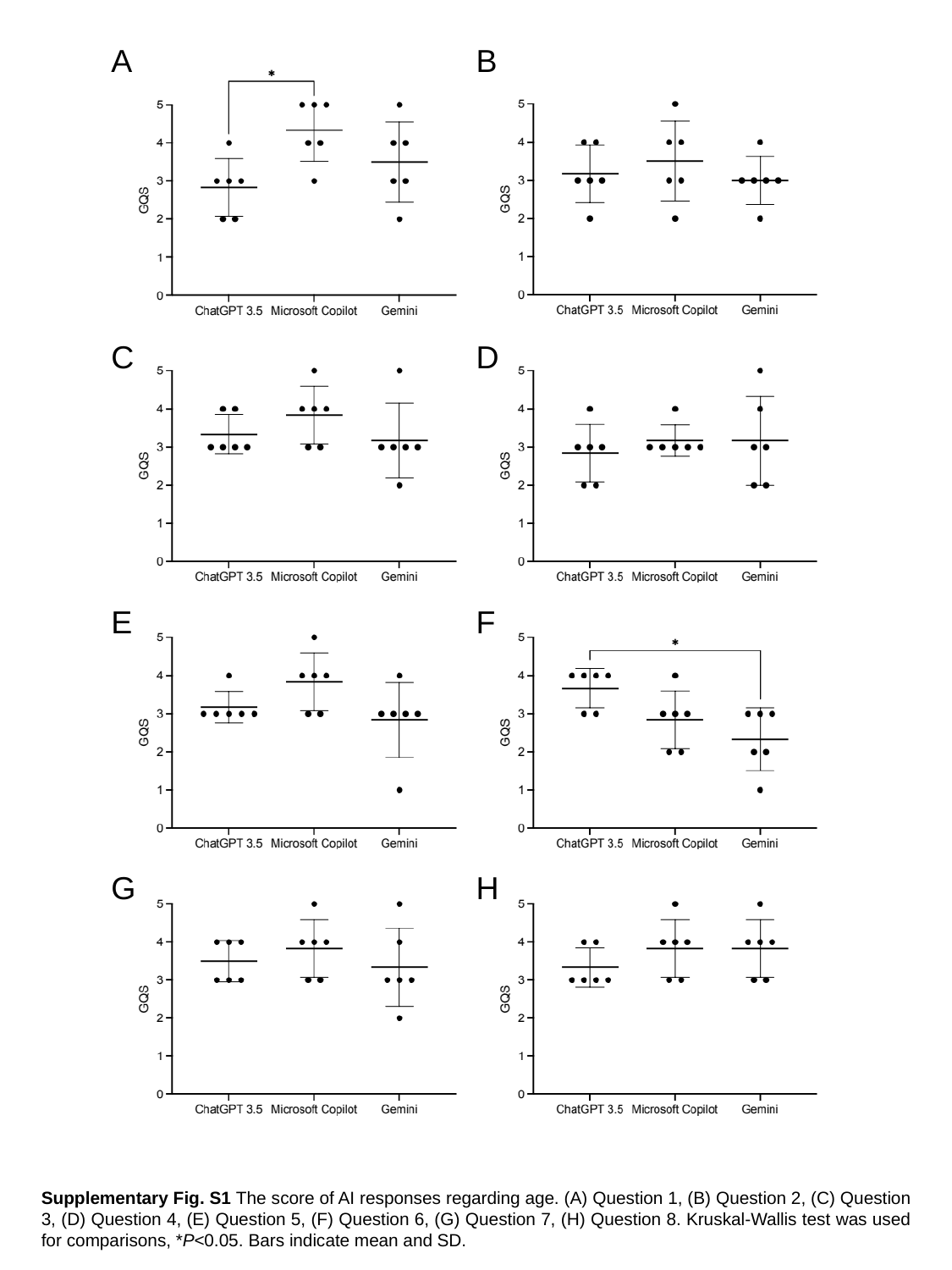

A
B
C
D
E
F
G
H
Supplementary Fig. S1 The score of AI responses regarding age. (A) Question 1, (B) Question 2, (C) Question 3, (D) Question 4, (E) Question 5, (F) Question 6, (G) Question 7, (H) Question 8. Kruskal-Wallis test was used for comparisons, *P<0.05. Bars indicate mean and SD.

## Slide 2
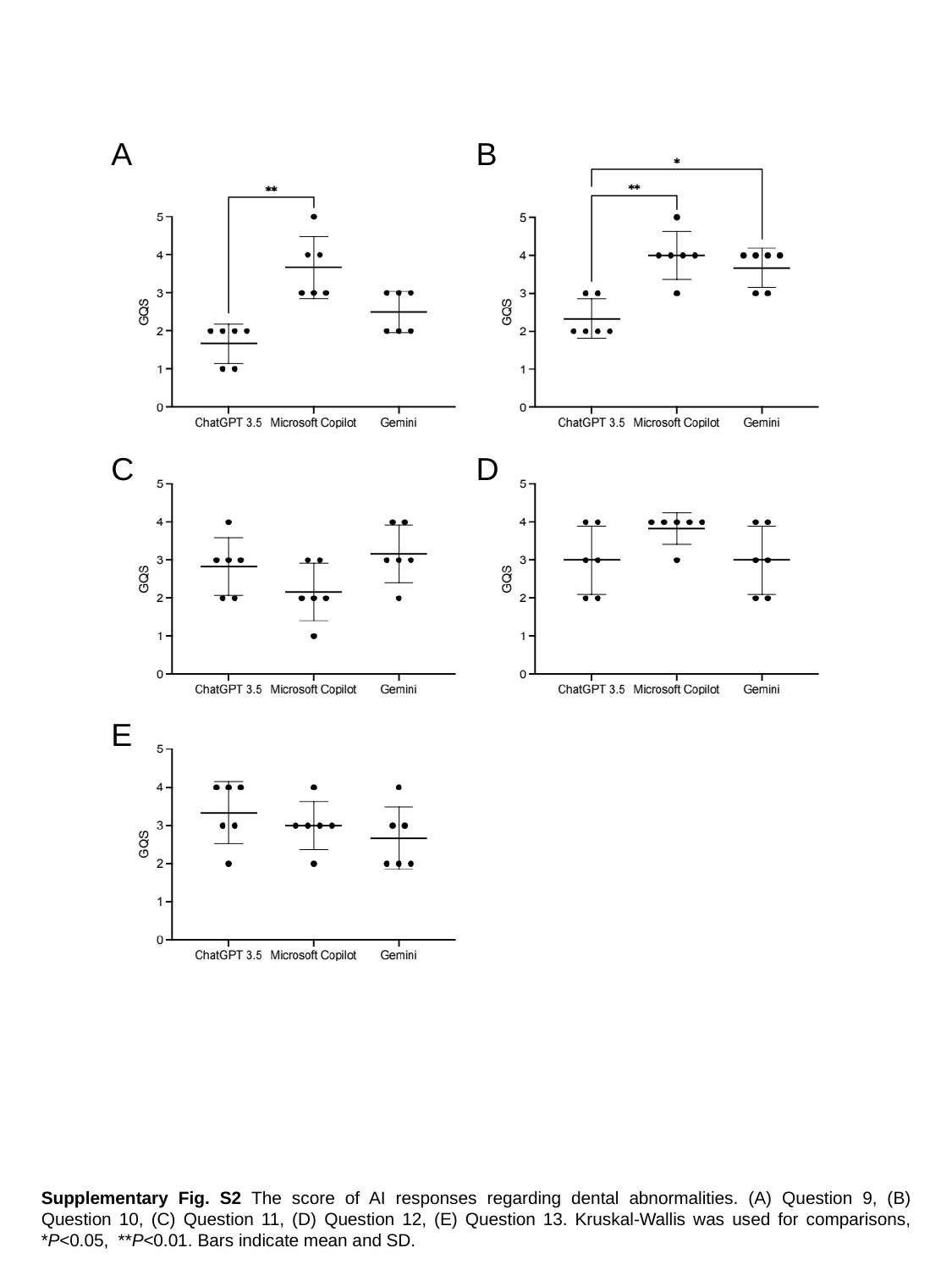

A
B
C
D
E
Supplementary Fig. S2 The score of AI responses regarding dental abnormalities. (A) Question 9, (B) Question 10, (C) Question 11, (D) Question 12, (E) Question 13. Kruskal-Wallis was used for comparisons, *P<0.05, **P<0.01. Bars indicate mean and SD.

## Slide 3
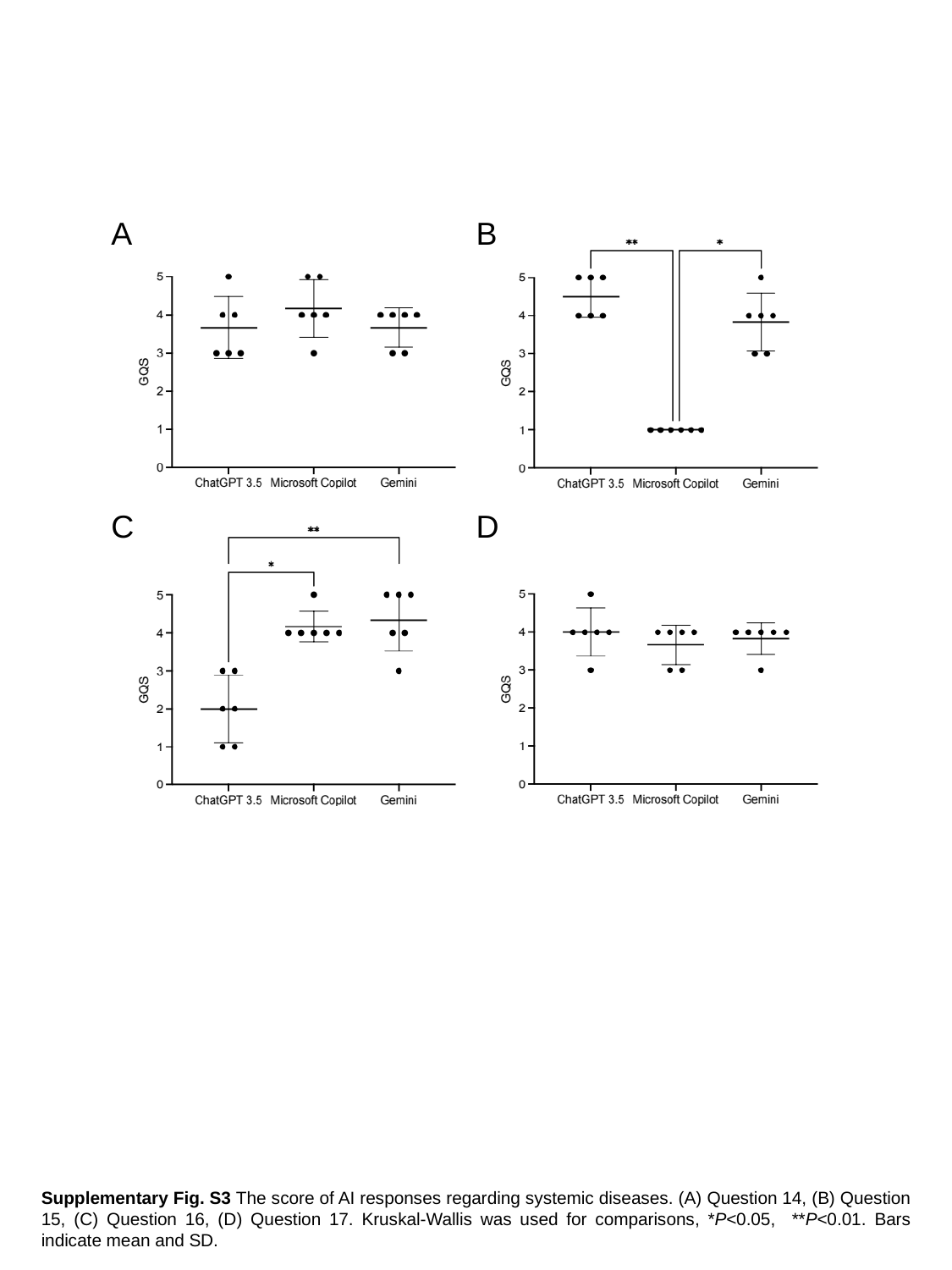

A
B
C
D
Supplementary Fig. S3 The score of AI responses regarding systemic diseases. (A) Question 14, (B) Question 15, (C) Question 16, (D) Question 17. Kruskal-Wallis was used for comparisons, *P<0.05, **P<0.01. Bars indicate mean and SD.

## Slide 4
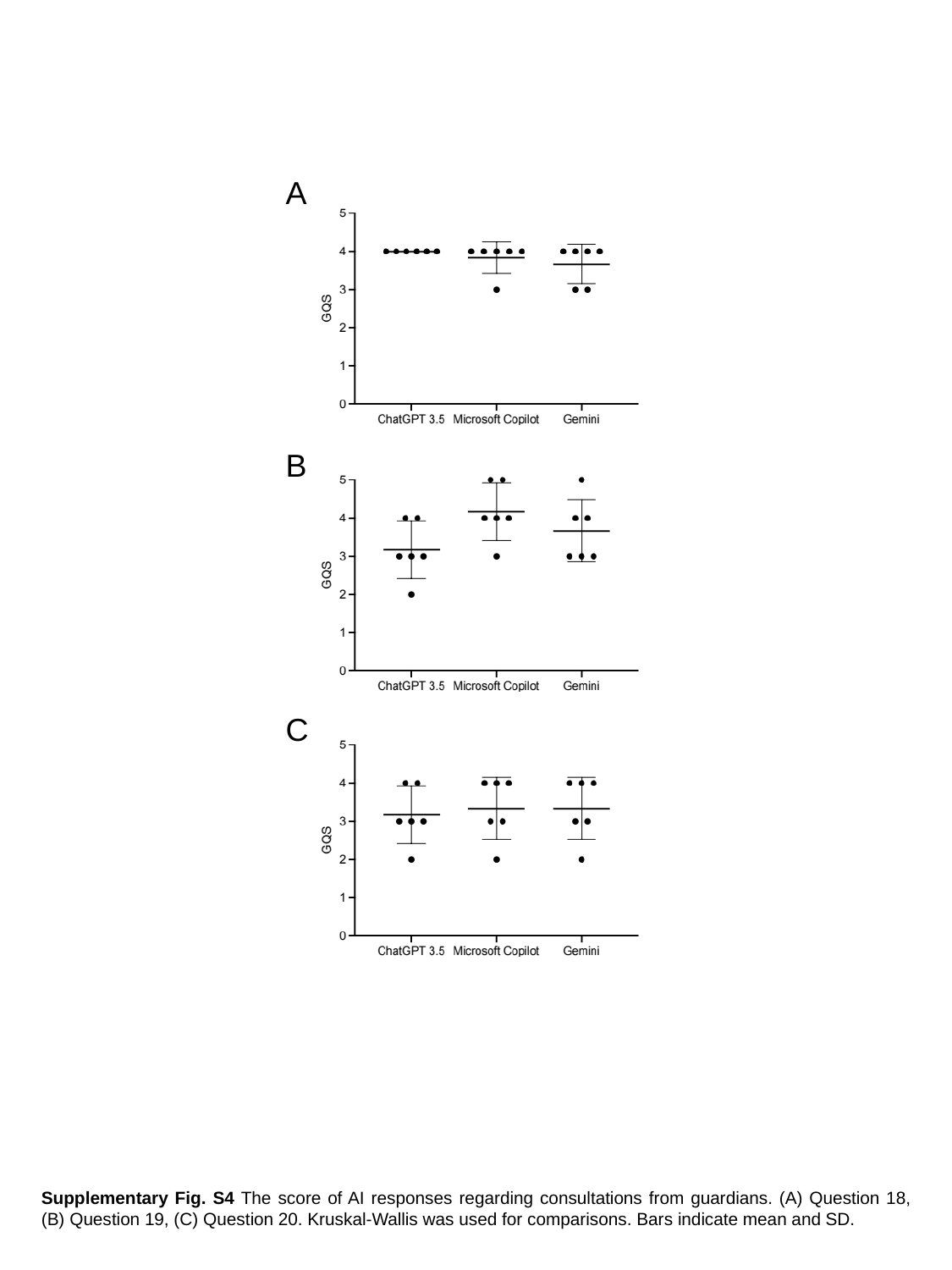

A
B
C
Supplementary Fig. S4 The score of AI responses regarding consultations from guardians. (A) Question 18, (B) Question 19, (C) Question 20. Kruskal-Wallis was used for comparisons. Bars indicate mean and SD.

## Slide 5
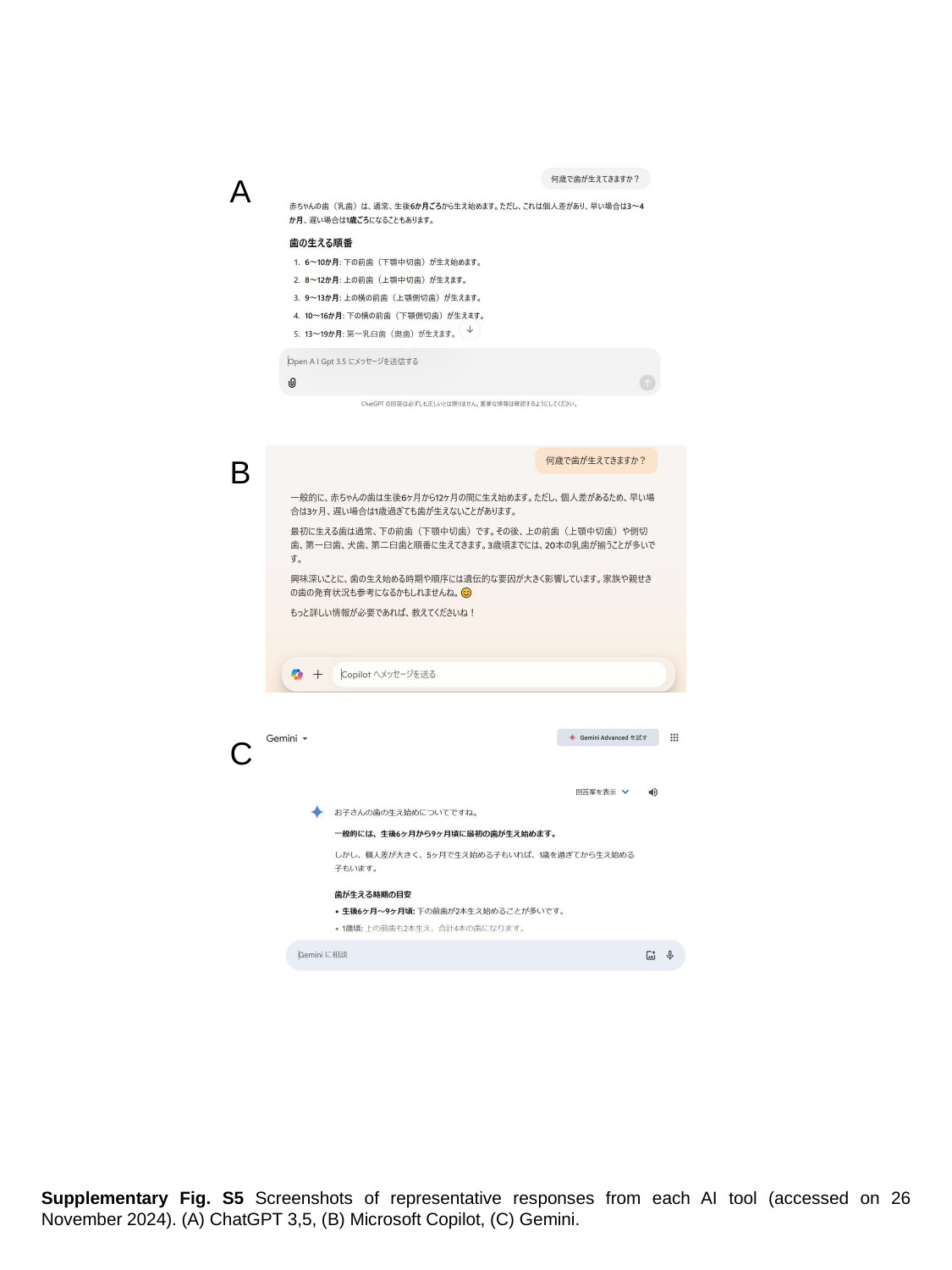

A
B
C
Supplementary Fig. S5 Screenshots of representative responses from each AI tool (accessed on 26 November 2024). (A) ChatGPT 3,5, (B) Microsoft Copilot, (C) Gemini.
